# The relationship of domestic pet ownership with the risk of childhood asthma: A systematic review and meta-analysis

**DOI:** 10.3389/fped.2022.953330

**Published:** 2022-07-22

**Authors:** Xiaoyi Ji, Yuan Yao, Ping Zheng, Chuangli Hao

**Affiliations:** ^1^Department of Respiratory Medicine, Children’s Hospital of Soochow University, Suzhou, China; ^2^Department of Pediatric, Jiaxing Maternal and Child Health Hospital, Jiaxing, China; ^3^Department of Radiology, Jiaxing Maternal and Child Health Hospital, Jiaxing, China

**Keywords:** domestic, pet ownership, cat, dog, childhood, asthma, severe, meta-analysis

## Abstract

**Background and Objective:**

The relationship between pet ownership and childhood asthma remains controversial. In recent years, there have been increasing studies with large sample size. Therefore, we conducted this systematic review and meta-analysis to evaluate the relationship between pet ownership and childhood asthma.

**Method:**

Relevant research was retrieved from PubMed, Cochrane, EMBASE, and Web of science. The retrieval was as of October 1, 2021. The Newcastle-Ottawa Scale (NOS) was used to assess the quality of the included studies. Stata 15.0 was used to conduct the meta-analysis.

**Results:**

A total of 60 studies with large sample size published between 1995 and 2021 were included in this systematic review and meta-analysis, which included 18 cohort studies and 42 case-control studies covering 27 countries and 1,871,295 children. As shown by meta-analysis results, cat ownership (OR = 1.18, 95%CI: 1.05∼1.33) and dog ownership (OR = 1.12, 95%CI: 1.0 0∼1.24) have a significant bearing on the occurrence of childhood asthma. Pet ownership was also positively correlated with the occurrence of severe childhood asthma (OR = 1.15, 95%CI: 1.11∼1.20).

**Conclusion:**

Pet ownership, especially cats and dogs, is associated with the occurrence of asthma in children.

## Introduction

Asthma is one of the most common chronic respiratory diseases in the world nowadays. The overall prevalence around the world still remains high in 2019. According to global statistics, in 2019, there were more than 300 million people suffering from asthma. Among them, there are about 30 million patients with asthma in China, among which children account for 6 million. The prevention and control of childhood asthma is a critical challenge (1). In recent years, the prevalence of childhood asthma has been increasing year by year. The occurrence of childhood asthma will definitely increase the burden on families and society, which makes it a serious public health issue.

As is well known, there are various risk factors related with the occurrence of asthma, including vitamin D deficiency, gastrointestinal and respiratory microbiota, early exposure to antibiotics, smoking, air pollution, genetic risk factors, stress, ethnic differences, etc. (2). Previous studies have shown that pet grooming is related to the occurrence of allergic rhinitis. Yang et al. (3) proposed in their study that keeping away from hairy pets can reduce the sensitization of children at high risk for allergic diseases. Moreover, Zhang et al. (4) pointed out that pet ownership, serving as a key risk factor for the incidence of air pollution in the crowd, is bound up with the occurrence of allergic rhinitis. The relationship between pet ownership and childhood asthma still remains controversial. The study by Takkouche et al. (5) demonstrated that exposure to furry pets such as cats and dogs was associated with asthma. A systematic review in 2012 involving 11 cohort studies including data from 22,000 children did not support that pet ownership in infancy was associated with the risk of asthma at 6-10 years of age (6).

In recent years, there has been increasing case-control or cohort studies with large sample size on the relationship between pet ownership and childhood asthma. Therefore, we conducted this systematic review and meta-analysis to investigate the relationship between pet ownership and childhood asthma to provide guidance on the prevention of childhood asthma (7–9).

## Method

### Search strategy

A comprehensive and systematic search was conducted by using PubMed, Cochrane, EMBASE, and Web of science databases. The retrieval was as of October 1, 2021. In order to avoid the risk of omitting original research published during our project, we continued searching the databases mentioned above until we completed this research. The region was not restricted in the search, and the retrieval method was subject headings + free words. The subject heading used in PubMed was asthma [Mesh], Child [Mesh], pet [Mesh]. The retrieval strategy used in PubMed is shown in supplementary document 1.

### Inclusion and exclusion criteria

The inclusion criteria were as follows: (1) The study type was case-control or cohort study. (2) The research subjects were children. (3) English literature. (4) The region and publication year were not limited. The exclusion criteria were as follows: (1) Non-English studies. (2) Studies including subjects older than children (>18 years old). (3) In order to avoid the influence brought by small sample size, we decided that studies with a sample size of less than 100 subjects should not be included in this systematic review. (4) Since the included original studies included case-control studies, there might be duplication of study subjects in multiple studies. Thus, if several studies contained the research subjects in the same region at the same time, we excluded studies with smaller sample size. (5) The confounding factors were not adjusted for outcome measures. (6) The original literature was not available.

### Literature screening and information extraction

The retrieved studies were imported into Endnote X9. After excluding duplication, the titles and abstracts were screened and eligible studies were downloaded. A comprehensive screening was performed to determine the included studies. Before information extraction, a spreadsheet of information extraction was prepared for this research, including title, author, publication year, country, study type, time of selecting the research subjects, source area of selecting the research subjects, types of pets, age of disease development, statistical method, and type of asthma and confounders. Our outcome indicator was presented as the odds ratio (OR) value after confounders were controlled.

The aforementioned information extraction was carried out independently by two researchers (Ji and Yao) and cross-checked after completion. If there was any dissent, a third researcher (Zheng) was consulted to assist in determination.

### Quality assessment

The types of the included original studies were cohort studies and case-control studies, and thus the Newcastle -Ottawa Scale (NOS) was used (10).

This scale evaluates the quality from three aspects: the selection of study groups, comparability between groups, and exposure/outcome. Among them, the highest scores in the selection of the study groups and the comparability between groups were 4 points and 2 points, respectively. If the exposure and outcomes were separately determined according to case-control study and cohort study, the highest score was 3 points. In the evaluation process, study with a score of >7 indicated high-quality research, and a score of 4-7 indicated moderate-quality research.

Quality assessment was conducted independently by 2 investigators (Ji and Yao) and cross-checked after completion. If there was any dissent, a third researcher (Zheng) was consulted to assist in determination.

### Statistical analysis

The statistics used in our study were OR and the 95% confidence interval (OR (95%CI)). The heterogeneity index, *I*^2^, was used to measure the heterogeneity of each study. When *I*^2^ ≥50%, a random-effects model was used. When *I*^2^ <50%, a fixed-effects model was used. To determine the sources of heterogeneity, subgroup analyses according to pet types was conducted.

The funnel plot was used to visually illustrae publication bias. Begg and Egger’s tests were used to perform statistical tests. If there was publication bias, the cut-and-fill method was used to analyze the impact of publication bias on this meta-analysis. All statistical analyses were performed using Stata version 15.0 (StataCorp LLC, College Station, TX). *P* < 0.05 indicated that the difference was statistically significant.

## Results

### Results of literature screening

**FIGURE 1:**
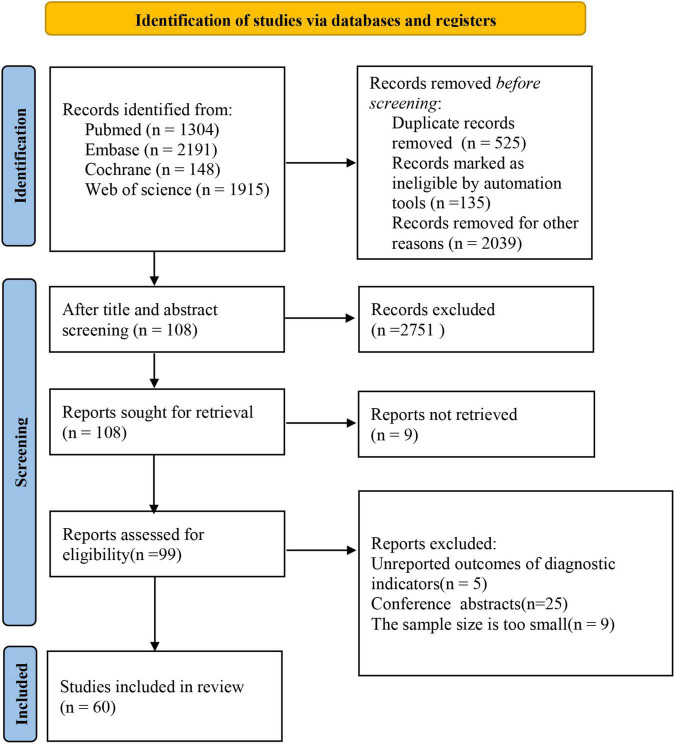
Flow chart of search process and reasons for exclusion.

Based on our search strategy, a total of 5,558 studies were retrieved. After excluding duplicates and step-by-step screening, 60 studies were finally included in this meta-analysis. The screening process is shown in Figure 1.

### Basic characteristics of the included studies

Among the 60 original studies, 18 were cohort studies (11–28) and 42 were case-control studies (7–9, 29–67), with 1,871,295 children included in this systematic review. The publication years of the included studies were from 1995 to 2021. The included studies covered 27 countries, with 26 from Europe, 15 from Asia, 12 from North America, 3 from Oceania, 3 from South America, and 1 from Africa. The detailed characteristics of the included studies were summarized in Table 1. In addition, the quality assessment results showed that most of the included studies were of high quality (Table 1).

**TABLE 1:** Characteristics of the included studies and the assessment of quality of the studies according to the Newcastle-Ottawa Scale (NOS).

Author	Author’s country	Year	Design	Sampling location	Sampling mechanism	Sampling time	Sample size	Age (year)	Gender (boy)	Animals	Score
Sabeti (63)	Iran	2021	Case control	Northwestern Iran	Areas urban	2019.11∼2020.02	1,459	14-18	791	U	7
Patra (59)	India	2021	Case control	Patna city in India	Schools	2015-2016	1,163	6-16	723	U	4
Park (27)	South Korea	2021	Cohort	located in Seoul	Medical centers and public-health centers	2007.11∼2015.12	1,163	0-7	597	Dogs	5
Ali (11)	Denmark	2021	Cohort	Denmark	Universities	2007-2015	327	0-14	278	Cats, Dogs	4
Zhang (9)	China	2020	Case control	Zhongshan, China	Schools	2016.03-07	1,1611	5-18	6087	Cats, Dogs, birds, Poultry	6
Ojwang (56)	Finland	2020	Case control	Turku, Oulu	Universities	1994-2004	3,781	0-5	1646	Dog	8
Morales-Romero (51)	Mexico	2019	Case control	Guadalajara, Mexico	Schools	2016.04∼06	1,992	15-18	936	Dog	4
AlShatti (7)	Kuwait	2020	Case control	The State of Kuwait	Schools	2016.09∼2017.05	3,864	11-14	1695	Cats, Dogs, birds, Poultry, Fish, Reptile	8
Simoneti (64)	Brasil	2018	Case control	Brazilian	universities	2017	191	2-4	60	Cats, Dogs	6
Hugg (41)	Finland	2008	Case control	Imatra, Finland and Svetogorsk, Russia	Schools	2003.10∼2003.11	1,093	7-14	517	Cats, Dogs, Rodent, Birds	8
McConnell (52)	California.	2002	Case control	in Southern California	Schools	The spring of 1998	3,535	9-16	1677	Cats, Dogs, Guinea pigs, Gerbils or hamsters Mice, Birds, other pets	7
Dong (34)	China	2008	Case control	Liaoning, China	Schools	2007.04	1,0784	9-11	5432	Cats, Dogs, Birds, Farm animals, other animals	7
Eldeirawi (36)	United States	2016	Case control	Chicago	Schools	2004.01∼05	1,816	4-18	881	Cats, Dogs, Birds	5
Nguyen (55)	United States	2010	Case control	New York	Communities	2002∼2003	1,412	0-17	748	U	6
Lindfors (48)	Sweden	1995	Case control	Sweden	Hospitals	1990.05∼1992.03	511	1-4	383	Cats, Dogs	7
Oluwole (57)	United States	2013	Case control	Nigeria	Communities and schools	2010-2017	1,736	13-14	785	Cat	5
Zheng (67)	China	2002	Case control	Beijing, China	Schools	1999.01∼2001.03	1,209	9-10	710	Cats, Dogs	7
Martel (24)	Canada	2009	cohort	Quebec, Canada	administrative health databases	1990∼2002	5,226	0-10	3041	U	8
Ernst (37)	Canada	1995	Case control	The island of Montreal	Schools	1993-1994	1,274	5-13	500	Cat	9
Strömberg Celind (28)	Sweden	2018	Cohort	Western Sweden	Hospitals	2003-2015	4,777	0-12	U	Cat	7
Pokharel (62)	Nepal	2001	Case control	South West of Delhi	Schools	1995.07∼1996.08	120	11-15	U	U	5
Collin (15)	United Kingdom	2014	Cohort	South-west England	Health Authority	1991.4.1∼1992.12.31	3,768	7.5–9	189	Any pet, Cat, Dog, Rabbit, Rodent, Bird	8
Milanzi (23)	Netherland	2019	Cohort	Netherlands.	Hospitals	1996-1997	1,871	0-17	926	U	7
Brunekreef (13)	Netherland	2012	Cohort	The world	Centers	2010-2012	53,5826	6-7	U	Cat, Dog	8
Yarnell (45)	Ireland	2003	Case control	Ireland	Schools	1995.03∼04	5,035	13-14	3029	Not dog or cat	8
Mitchell (25)	New Zealand	2007	Cohort	New Zealand	Hospitals	1995.10∼1997.11	871	1-7	310	Dog	7
Oberle (58)	Germany	2003	Case control	Southern Babaria	Schools	1997	8,216	5-7	U	Cat	9
Waser (66)	Switzerland	2005	Case control	Rural areas of Austria, Germany and Switzerland	Schools	1999.03-07	812	9-11	414	Cat, Dog	9
Bcklund (12)	Sweden	2006	Cohort	Northern Sweden	Schools	1996-2000	3,525	7–12	758	Cat, Dog	9
Kerkhof (21)	Netherlands	2009	Cohort	Netherlands	Prenatal clinics	1996–1997	2,951	0-8	1531	Cat, Dog	9
Hölscher (42)	Germany	2002	Case control	Zerbst and Hettstedt areas	Schools	1992-1999	7,448	5-14	3902	Cat, Dog	7
Garcia (39)	America	2008	Case control	Bogota’, Colombia	Schools	2002.03-09	7,085	6-14	3237	Cat, Dog	7
Chan-Yeung (16)	Canada	2008	Cohort	Winnipeg and Vancouver, Canada	Centers	1995-2002	380	0-7	200	Cat, Dog	8
Naydenov (53)	Denmark	2008	Case control	Bulgaria	Towns	2004	4,479	2-7	U	Cat, Dog	7
Lombardi (46)	Italy	2010	Case control	Italy	Schools	2002	2,0016	0-7	10288	Dog	7
Garrett (20)	Australia	1998	Cohort	Australia	Schools	1994.03-1995.02	148	7-14	U	U	6
Dong (35)	China	2009	Case control	Liaoning, China	Schools	2007.04	1,2910	7-13	6416	Cat, Dog, Bird, farm animals, other animals	7
Bertelsen (14)	Norway	2010	cohort	Oslo	Hospitals	1992-1993	1,019	0-10	551	Cat, Dog, other furry pets	7
Morass (50)	Austria	2007	Case control	Tyrol	Schools	2006	1,761	5-7	U	U	6
Janson (43)	Sweden	2007	Case control	Uppsala, Sweden	Schools	1998.10-1999.12	959	13-14	805	Cat or dog	5
Neto (54)	Brazil	2019	Case control	Passo Fundo	Schools	2009.03-2011.12	1,003	9-11	377	Cat	4
de Marco (32)	Italy	2004	Case control	Europe, North America, and Oceania.	Centers	1991-1993	1,8156	0-10	8683	Cat	9
Grabenhenrich (19)	Germany	2014	Cohort	Germany	Schools	1990.01-12	1,314	0-14	494	Cat, Dog	9
O’Connor (26)	United States	2017	Cohort	Baltimore, Boston, New York City, and St Louis.	Hospitals	2005.02-2007.03	442	0-7	226	Cat, Dog	9
Luo (8)	China	2017	Case control	Tianjin, Chi	Schools	2013.04	7,366	0-8	3780	Cat, Dog, Rodent, Bird, Fish	7
Dong (33)	China	2008	Case control	Liaoning China	Schools	2007.04	1,4729	4-6	7409	Cat, Dog, Bird, et al	5
Fall (18)	Sweden	2015	cohort	Sweden	Hospitals	2001.1.1-2010.12.31	1,011051	0-10	U	Dog, farm animals	7
Sasaki (65)	Japan	20n15	Case control	Japan	Center	2012.06	3,066	8-10	1818	U	7
Ardura-Garcia (30)	United States	2015	Case control	Ecuador	Hospitals	2012.10-12	179	5-15	94	U	8
Medjo (49)	Serbia	2013	Case control	Belgrsde, Serbia	Hospitals	2012	149	7-14	90	Cat, Dog	4
Lanphear (47)	United States	2001	Case control	United States	Househoods	1988-1994	8,257	6-16	4086	dog	8
Ayuk (29)	South Africa	2018	Case control	South Africa	Schools	2001.01-2002.11	28,391	13-14	13965	Cat, Dog	7
Perzanowski (61)	Swedish	2002	Case control	Sweden	Schools	1996	3,431	7-8	1749	Cat, Dog	9
Arnedo-Pena (31)	Spain	2009	Case control	Castellon (Spain)	Schools	2002	4,492	6-7	2220	Cat, Dog	4
Janahi (44)	Qatar	2006	Case control	Qatar	Schools	2003.02-2004.02	3,500	6-14	U	U	4
Furuhata (22)	Japan	2020	Cohort	Japan	Hospitals	2001.1.10-27	45,060	1-10	23047	U	5
Chen (40)	China	2013	Case control	Shanghai, China	schools	2011.04-2012.04	13,335	4-6	6753	Cat, Dog, Rodent, et al	6
Fall (17)	Sweden	2018	Cohort	Swedish	Hospitals	2001.01-2004.12	23,585	1-6	U	Dog	7
Palvo (60)	Brazil	2008	Case control	SJRP	schools	2003.11-2004.10	2,206	6-7	U	U	5
Fedortsiv (38)	Ukraine	2012	Case control	Ternopil	schools	2010	4,871	6-14	2396	U	7

### Meta-analysis results

#### The overall risk analysis on pet ownership and the onset of childhood asthma

**FIGURE 2:**
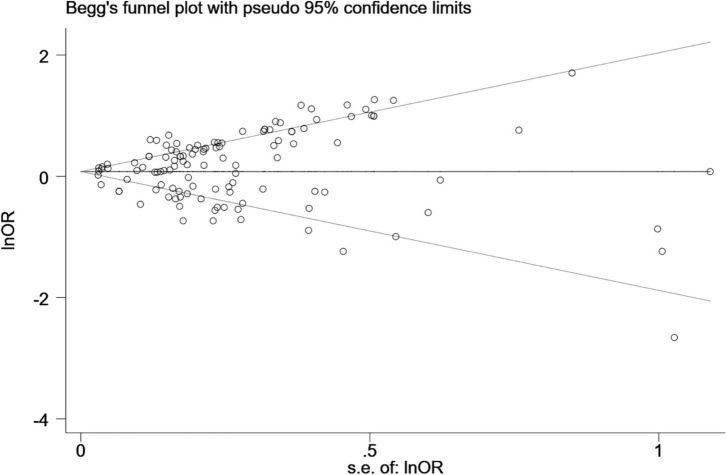
Begg’s funnel plot of the association between pet ownership and childhood asthma.

A random-effects model (*I*^2^ = 79.1%) was used for the meta - analysis on 60 included studies. The results showed that the pet ownership was significantly correlated with the risk of childhood asthma onset (OR = 1.14, 95%CI: 1.08-1.21). Supporting information included the results of sensitivity analysis and publication bias assessment (Figure 2) (*P* < 0.05).

#### Subgroup analysis

**FIGURE 3:**
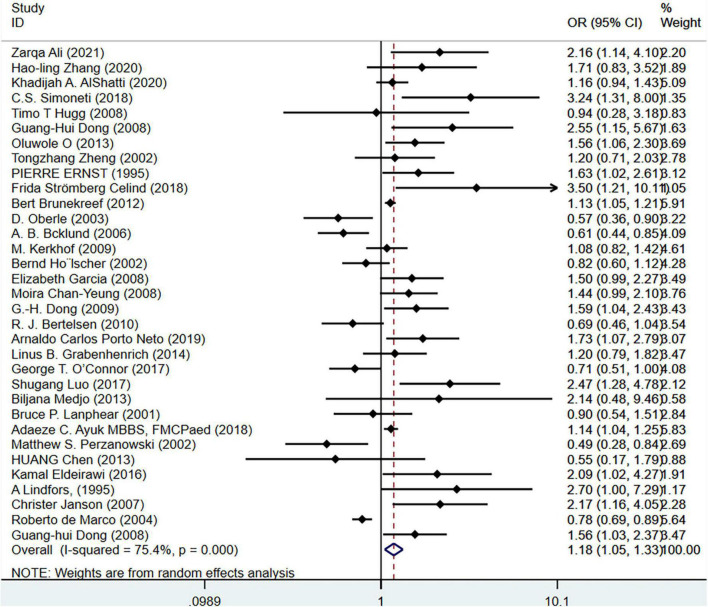
Forest plot of the association between cat ownership and childhood asthma.

**FIGURE 4:**
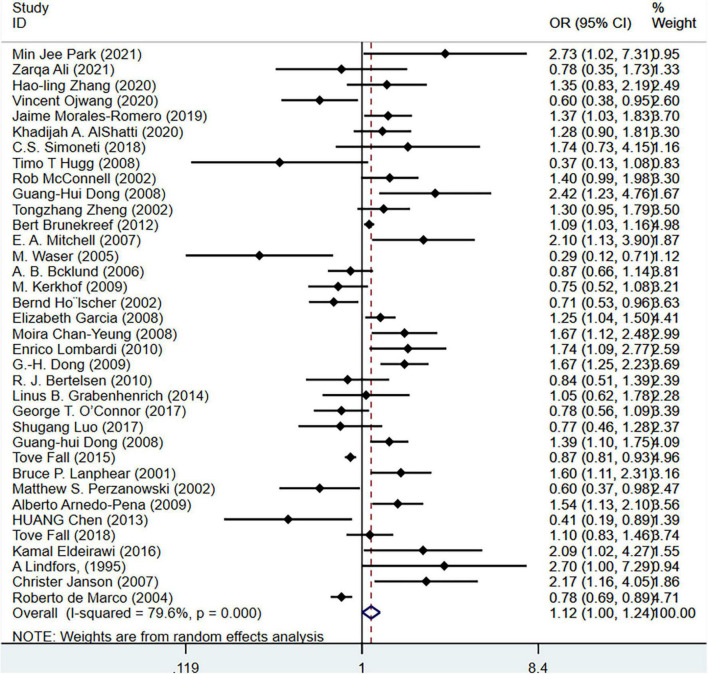
Forest plot of the association between dog ownership and childhood asthma.

**FIGURE 5:**
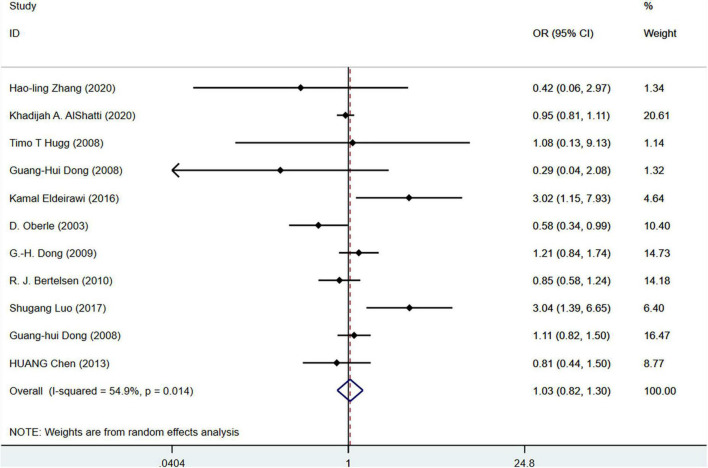
Forest plot of the association between furry pet ownership and childhood asthma.

**FIGURE 6:**
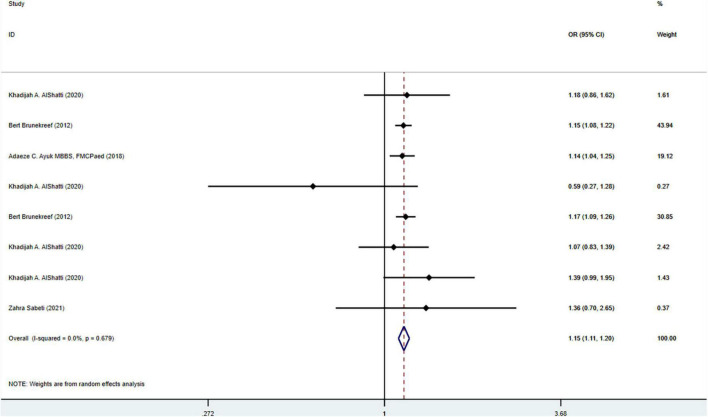
Forest plot of the association between pet ownership and severe childhood asthma.

First, we performed a subgroup analysis based on pet types. The results of the meta-analysis on 33 studies (*N* = 716,085) showed that cat ownership is significantly related to the occurrence of childhood asthma (OR = 1.18, 95%CI: 1.05-1.33, *I*^2^ = 75.4%, Figure 3). The results of the meta-analysis on 36 studies (*N* = 1,728,907) showed that dog ownership is significantly associated with the occurrence of childhood asthma (OR = 1.12, 95%CI: 1.00-1.24, *I*^2^ = 79. 6%, Figure 4). The results of the meta-analysis on 11 studies (*N* = 86,743) showed that other furry pet ownership was not associated with the occurrence of childhood asthma (OR = 1.03, 95%CI: 0.82-1.30, *I*^2^ = 54.9%, Figure 5). This study takes samples mainly from schools (OR = 1.223, 95%CI:1.012∼1.480,*I*^2^ = 77.4%) and hospitals (OR = 1.132, 95%CI:1.040∼1.233,*I*^2^ = 79.0%). Only few studies conducted subgroup analysis by gender, so it is insufficient to conduct a subgroup analysis by gender in our study. In addition, our study demonstrated that pet ownership was also significantly associated with severe childhood asthma (OR = 1.15, 95%CI: 1.11-1.20, *I*^2^ = 67.9%, Figure 6).

## Discussion

This systematic review and meta-analysis on a large number of original studies with quantities of observational samples presented that pet ownership was significantly correlated with the risk of childhood asthma. There was a clear relationship between cat and dog ownership and the risk of childhood asthma. In addition, pet ownership was associated with the risk of severe childhood asthma. These results were obtained after pooling individual studies and adjusting the confounders.

Bronchial asthma is the most common chronic airway inflammatory disease in children. In recent years, the incidence of this disease has been increasing in our country, and asthma has become a public health issue attracting global attention. The etiology of asthma is complicated and closely related to genetic background, environmental factors, and immune function, including vitamin D deficiency, gastrointestinal and respiratory microbiota, early exposure to antibiotics, smoking, air pollution, genetic risk factors, stress, ethnic differences, etc. (2).

Cats and dogs have been companions for humans for thousands of years. Pet ownership provide various health benefits for humans, including ameliorating loneliness, attenuating psychological changes, promoting relationships, and acting as a protective factor against certain diseases, such as cardiovascular disease (68). In spite of these advantages, our study found that pet ownership, especially cats and dogs, could increase the risk of childhood asthma.

Currently, the mechanism underlying the positive correlation betweeen pet ownershipand the risk of childhood asthma remains unclear. Herre et al. (69) found that cats could secrete a type of protein, Feld 1, which was similar to Derp 2, the allergen in dust mites. Feld 1 can enhance signaling through innate receptors TLR4 and TLR2. The allergen from dogs, Canf 6, is a type of lipoprotein allergens with similar charateristics to Feld 1. Studies believed that Feld 1 and Canf 6 belonged to a group of allergen immunomodulatory proteins which significantly amplified LPS/TLR signaling in macrophage-like cells, thereby enhancing innate immune signaling and promoting airway hypersensitivity in patients with asthma. Other studies have found (70, 71) that pet is the main source of household endotoxins. Endotoxins are considered as pro-inflammatory agents which may be associated with promoting asthma (71, 72). A study by Akramiene et al. (73) and Douwes et al. (74) found that pet ownership increased the indoor concentration of fungi and dust. β-(1, 3)-d-glucan (beta-glucan), a glucose polymer existing in the fungal cell wall, was positively associated with the variability of peak expiratory flow (PEF) in children with asthma. Maheswaran et al.(75) discovered that exposure to β-glucan was a risk factor for atopic asthma. β-glucan can induce DC to stimulate Th17 cells, and the activation of Th17 cells may trigger inflammatory responses.

In recent years, there has been increasing case-control or cohort studies with large sample size on the relationship between pet ownership and childhood asthma. A study by Morales-Romeroden et al. (51) found that exposure to cats and dogs increased the risk of childhood asthma. Ayuk et al. (29) found that cat ownership is associated with severe asthma after investigating 258,267 children aged 13-14 in 10 African centers. Eldeirawi et al. (36) discovered that exposure to such pets as cats and dogs *in utero* is linked to an increased chance of asthma when studying Mexican American children aged 4-18. In the study of the relationship between bronchial hyperreactivity and the occurrence of asthma in children at the age of 7, Park et al. (27) found that dog ownership significantly increases the risk of non-atopic asthma. In 2020, in a survey on 11,611 students in Zhongshan City, China, Zhang et al. (9) found that pet ownership was associated with the prevalence of childhood asthma and the occurrence of asthma-related symptoms, especially in those who owned cats, poultry or slept with pets. A study in Tianjin demonstrated that (8), exposure to pets at an early age was positively correlated with asthma in Tianjin. A survey of 12 districts in 3 cities in Liaoning Province presented that pet ownership increased the risk of childhood asthma. In a cohort study by Ali et al. (11) found that pet ownership was positively related with the prevalence of asthma. In a population-based birth cohort study by Strömberg Celind et al. (28), it was also discovered that cat ownership increased the risk of uncontrolled asthma. The findings of these studies were consistent with our results on the association between pet ownership and childhood asthma, suggesting that our conclusion was reliable. Sensitivity analysis also proved the reliability of our results.

There were some advantages in our study. First, our research was based on a large number of studies with large sample size, which ensured the level of evidence. Second, the time of the studies included in our research was continuous, from 1995 to 2021. There will be literature reports in 2021. Third, the included studies involved all regions around the world, which weakened the influence brought by environment, race, and attitudes to a certain extent. Fourth, there was no publication bias in our research. Last, this research was the first proposing the association between pet ownership and severe asthma. Nonetheless, there were still some limitations. First, limited to the information in the original studies, we could not summarize the relationship between pet ownership and risk of childhood asthma according to different age groups. Second, limited to the information in the original studies, we were unable to obtain detailed information on the types of pets. Third, it was impossible to quantify and compare the risk of asthma in children with various pets other than cats and dogs.

## Conclusion

We found that pet ownership, especially cats and dogs, was positively correlated with the occurrence of childhood asthma. Pet ownership was also positively correlated with the occurrence of severe childhood asthma. However, we did not deny human’s love for pet. When raising pets, it is better to choose those with less impact on the occurrence of childhood asthma. Furthermore, the contact of pets with infants should be avoided. At the same time, hygiene management of pets requires to be enhanced.

## Data availability statement

The original contributions presented in this study are included in the article/supplementary material, further inquiries can be directed to the corresponding author/s.

## Author contributions

XJ, YY, and CH contributed to the conception and design of the study. YY organized the database. PZ performed the statistical analysis. XJ wrote the first draft of the manuscript. YY, PZ, and CH wrote sections of the manuscript. All authors contributed to manuscript revision, read, and approved the submitted version.

## Conflict of interest

The authors declare that the research was conducted in the absence of any commercial or financial relationships that could be construed as a potential conflict of interest.

## Publisher’s note

All claims expressed in this article are solely those of the authors and do not necessarily represent those of their affiliated organizations, or those of the publisher, the editors and the reviewers. Any product that may be evaluated in this article, or claim that may be made by its manufacturer, is not guaranteed or endorsed by the publisher.
